# Increased Plasma Soluble Interleukin-2 Receptor Alpha Levels in Patients With Long-Term Type 1 Diabetes With Vascular Complications Associated With *IL2RA* and *PTPN2* Gene Polymorphisms

**DOI:** 10.3389/fendo.2020.575469

**Published:** 2020-10-30

**Authors:** Magdalena Keindl, Olena Fedotkina, Elsa du Plessis, Ruchi Jain, Brith Bergum, Troels Mygind Jensen, Cathrine Laustrup Møller, Henrik Falhammar, Thomas Nyström, Sergiu-Bogdan Catrina, Gun Jörneskog, Leif Groop, Mats Eliasson, Björn Eliasson, Kerstin Brismar, Peter M. Nilsson, Tore Julsrud Berg, Silke Appel, Valeriya Lyssenko

**Affiliations:** ^1^ Center for Diabetes Research, Department of Clinical Science, Faculty of Medicine, University of Bergen, Bergen, Norway; ^2^ Broegelmann Research Laboratory, Department of Clinical Science, Faculty of Medicine, University of Bergen, Bergen, Norway; ^3^ Department of Clinical Science, Lund University Diabetes Centre, Malmö, Sweden; ^4^ Flow Cytometry Core Facility, Department of Clinical Science, Faculty of Medicine, University of Bergen, Bergen, Norway; ^5^ Research Unit for General Practice & Danish Ageing Research Center, Department of Public Health, University of Southern Denmark, Odense, Denmark; ^6^ Clinical Epidemiology, Steno Diabetes Center Copenhagen (SDCC), Gentofte, Denmark; ^7^ Translational Pathophysiology, Steno Diabetes Center Copenhagen (SDCC), Gentofte, Denmark; ^8^ Department of Molecular Medicine and Surgery, Karolinska Institute, Stockholm, Sweden; ^9^ Department of Endocrinology, Metabolism and Diabetes, Karolinska University Hospital, Stockholm, Sweden; ^10^ Department of Clinical Science and Education, Division of Internal Medicine, Unit for Diabetes Research, Karolinska Institute, South Hospital, Stockholm, Sweden; ^11^ Center for Diabetes, Academica Specialist Centrum, Stockholm, Sweden; ^12^ Karolinska Institute, Department of Clinical Sciences, Danderyd University Hospital, Division of Internal Medicine, Stockholm, Sweden; ^13^ Institute for Molecular Medicine Finland (FIMM), University of Helsinki, Helsinki, Finland; ^14^ Department of Public Health and Clinical Medicine, Sunderby Research Unit, Umeå University, Umeå, Sweden; ^15^ Department of Medicine, University of Gothenburg, Gothenburg, Sweden; ^16^ Institute of Clinical Medicine, Faculty of Medicine, University of Oslo, Oslo, Norway

**Keywords:** cardiovascular disease, Cluster of Differentiation 25 (CD25), diabetes complications, nephropathy, regulatory T cells, retinopathy, sIL-2R

## Abstract

Type 1 diabetes (T1D) is largely considered an autoimmune disease leading to the destruction of insulin-producing pancreatic β cells. Further, patients with T1D have 3–4-fold increased risk of developing micro- and macrovascular complications. However, the contribution of immune-related factors contributing to these diabetes complications are poorly understood. Individuals with long-term T1D who do not progress to vascular complications offer a great potential to evaluate end-organ protection. The aim of the present study was to investigate the association of inflammatory protein levels with vascular complications (retinopathy, nephropathy, cardiovascular disease) in individuals with long-term T1D compared to individuals who rapidly progressed to complications. We studied a panel of inflammatory markers in plasma of patients with long-term T1D with (n = 81 and 26) and without (n = 313 and 25) vascular complications from two cross-sectional Scandinavian cohorts (PROLONG and DIALONG) using Luminex technology. A subset of PROLONG individuals (n = 61) was screened for circulating immune cells using multicolor flow cytometry. We found that elevated plasma levels of soluble interleukin-2 receptor alpha (sIL-2R) were positively associated with the complication phenotype. Risk carriers of polymorphisms in the *IL2RA* and *PTPN2* gene region had elevated plasma levels of sIL-2R. In addition, cell surface marker analysis revealed a shift from naïve to effector T cells in T1D individuals with vascular complications as compared to those without. In contrast, no difference between the groups was observed either in IL-2R cell surface expression or in regulatory T cell population size. In conclusion, our data indicates that *IL2RA* and *PTPN2* gene variants might increase the risk of developing vascular complications in people with T1D, by affecting sIL-2R plasma levels and potentially lowering T cell responsiveness. Thus, elevated sIL-2R plasma levels may serve as a biomarker in monitoring the risk for developing diabetic complications and thereby improve patient care.

## Introduction

Type 1 diabetes (T1D) is characterised by T cell mediated autoimmune destruction of insulin-producing pancreatic β cells (1, 2), causing T1D individuals to become insulin-dependent throughout their life (3). Most of the patients with T1D develop macrovascular complications such as cardiovascular disease (CVD), and microvascular complications, including proliferative diabetic retinopathy (PDR), chronic kidney disease (CKD), and peripheral neuropathy (PN) (4). The rising prevalence of T1D and its associated long-term vascular complications clearly confer a humanistic (5) and socio-economic burden (6). Vascular complications in T1D individuals are a common cause of mortality related to end-organ damage as compared to the non-diabetic population (4, 7). Remarkably, few patients with T1D do not progress to these vascular complications despite long disease duration and chronic hyperglycaemia, and therefore exert great potential to evaluate end-organ protection (8).

Although T1D individuals with complications show considerable derangement in immunological processes like having elevated concentrations of C-reactive protein (CRP), a marker of inflammation, proinflammatory cytokines interleukin-6 (IL-6), and tumor necrosis factor alpha (TNF-α) as compared to individuals without complications (9), the extent of contribution of immunological factors to the development of vascular complications in patients with T1D is poorly understood.

Over the past decades, both genetic (10, 11) and immunological (12, 13) studies revealed IL-2 receptor (IL-2R) and its downstream signalling pathways as central players in the pathogenesis of T1D (14). Upon binding of IL-2 to its receptor IL-2R, a cascade of signalling events is initiated. These events are negatively regulated by the ubiquitously expressed phosphatase tyrosine-protein phosphatase non-receptor type 2 (PTPN2) (14, 15). IL-2 signalling is critical for the function of regulatory T cells (Tregs), a type of suppressive immune cell, which plays an indispensable role in maintaining immune homeostasis (16) and prevention of autoimmune diseases (17, 18). In addition, elevated levels of soluble IL-2R alpha (sIL-2R; alternative: IL-2RA, CD25) have been reported to be an important factor in the development of diabetic retinopathy in non-insulin-dependent diabetes patients (19) and coronary artery calcification in T1D patients (20). However, a limitation of both studies was that the patient group without complications had a considerably shorter diabetes duration compared to the patient group with the respective complications. Therefore, some of the patients in the complications-free group could have later progressed to vascular complications. Furthermore, Colombo et al. (2019) reported that elevated levels of sIL-2R were associated with a decline in estimated glomerular filtration rate (eGFR) in T1D patients (21).

The aim of the present study was to evaluate plasma levels of inflammatory proteins including but not limited to sIL-2R in long-term T1D individuals with and without vascular complications. Additionally, a subset of patients was screened for circulating immune cells to investigate cell populations associated with developing vascular complications in T1D individuals. Finally, plasma sIL-2R levels were correlated to genetic risk variants in *IL2RA* and *PTPN2*.

## Methods

### Study Design

This immunological investigation forms a part of the PROtective genetic and non-genetic factors in diabetic complications and LONGevity (PROLONG) study, which was launched to identify protective factors against complications in long-term T1D individuals. Patients were recruited from seven departments of endocrinology/diabetes outpatient clinics in Sweden and at the Steno Diabetes Center in Denmark. As an extended collaborative effort, we included T1D individuals from a Norwegian cohort, DIALONG. The DIALONG study also included non-diabetic spouses or friends as a control group.

We classified T1D patients into two groups comparing their diabetes complications status. Non-progressors (NP) were defined as patients with a T1D duration of over 30 years without having progressed to any of the specified complications. Patients who progressed to complications within 25 years of T1D diagnosis were defined as rapid progressors (RP). We defined late progressors (LP) as T1D patients that did progress to complications >25 years post diagnosis. For this study RPs and LPs were pooled into one group referred to as progressors (P).

In PROLONG, PDR was defined by the presence of proliferative retinopathy in at least one eye, confirmed laser treatment (panretinal photocoagulation) or blindness. For CKD we used the following inclusion criteria: presence of nighttime albuminuria over 200 µg/min, macroalbuminuria over 300 mg/g or a documented diabetic kidney disease diagnosis. None of the PROLONG participants were treated with SGLT2 inhibitors. Documented events including non-fatal myocardial infarction, stroke (haemorrhagic or ischemic), balloon angioplasty, or coronary artery bypass were defined as CVD. There was limited information on CVD, as the original focus of PROLONG was on microvascular complications.

In DIALONG (22) macro- and microvascular complications were defined using similar criteria as in PROLONG. PDR was defined by the presence of proliferative retinopathy or blindness. Laser treatment was not used as criteria here as it was not exclusively applied to proliferative retinopathy in this study. CKD was adjusted to include patients with persistent microalbuminuria or proteinuria. None of the DIALONG participants were treated with SGLT2 inhibitors. CVD was defined by stroke or myocardial infarction events, coronary artery bypass or percutaneous coronary intervention (PCI), diagnosed peripheral vascular disease or heart failure.

The regional ethical committees approved the studies (PROLONG: Denmark H-2-2013-073, Sweden 777/2009, Norway 2019/1324; DIALONG: Norway 2014/851) and all participants provided written informed consent.

### Blood Sampling

Collected EDTA plasma was aliquoted and stored at -80°C for ~6 years (PROLONG) and ~3 years (DIALONG).

For peripheral blood mononuclear cells (PBMCs) isolation, peripheral blood from patients with T1D (PROLONG) with and without complications was collected in CPT tubes (BD Vacutainer) at the Steno Diabetes Center, Denmark. PBMCs were subsequently isolated by density gradient centrifugation and cryopreserved in human AB serum containing 10% DMSO at -80°C for ~5 years.

### Cytokine Analysis

In DIALONG, plasma cytokines were measured in 79 individuals using an Invitrogen™ Human Cytokine 30-Plex kit (LHC6003M, Thermo Fisher Scientific) according to the manufacturer’s instructions. The following adjustments were made to the protocol: (a) one additional standard was included in the serial dilution, making the standard range from 1:3 to 1:2,187; (b) undiluted plasma samples that underwent one freeze-thaw cycle were measured. The following biomarkers were detected in >90% of samples (CCL11, IFN-α, IL-12, sIL-2R, CXCL10, CCL2, CCL3, CCL4 and CCL5), whereas others were only detected in 40–75% (FGF basic, G-CSF, HGF, IL-13, IL-1RA, CXCL9, VEGF) and <17% of patients (EGF, IFN-γ, IL-10, IL-15, IL-17A, IL-1β, IL-2, IL-4, IL-6, TNF-α). GM-CSF, IL-5, IL-7, and IL-8 were not detected in any samples.

In PROLONG, we used a custom-designed ProCartaPlex™ Human Cytokine Panel (sIL-2R, CCL2, CCL11, IFN-α; Thermo Fisher Scientific) according to manufacturer’s instructions to measure plasma cytokines. The following adjustments were made to the protocol: (a) one additional standard was included in the serial dilution, making the standard range from 1:4 to 1:32,768 (in pg/ml: sIL-2R 10.06–82,425; CCL2 0.45–3,650; CCL11 0.07–550; IFN-α 0.08–625); (b) undiluted plasma samples that underwent one freeze-thaw cycle were measured.

Data was acquired on a Luminex^®^ 100™, counting 3,000 (DIALONG) and 600 (PROLONG) beads per well. The five-parameter logistic algorithm [weighted by 1/y, (V2.4)] and raw median fluorescence intensity values were used for the creation of standard curves.

### Genetic Analysis

The DNA samples in the PROLONG study were genotyped using InfiniumCoreExome-24v1-1 array. Standard quality control steps for GWAS were performed. Imputation was performed using Michigan Imputation Server (https://imputationserver.sph.umich.edu/index.html) using Haplotype Reference Consortium Release 1.1 (HRC.r1-1, GRCh37) as a reference panel. Variants with minor allele frequency (MAF) <5% were excluded before imputation. In the present study, we extracted the region for the *IL2RA* (Chromosome 10: 6,052,652-6,104,288, build GRch37) and *PTPN2* gene (Chromosome 18: 12,785,477-12,929,642, build GRch37) for analysis. Only variants with imputation quality R^2^ >0.4 and with MAF >5% were included in the analysis.

Principal component analysis (PCA) was performed on pruned, directly genotyped SNPs using 1,000 Genomes’ reference panel version 5A. Population outliers were examined based on visual inspection of PC1 and PC2, and outliers were excluded from the further analysis. Only individuals with complete data on sIL-2R, sex, complication group, HbA1c and age at visit were included. In total, there were 330 individuals analyzed. We used linear regression adjusted for sex, age, and complication group to identify associations between genetic variants and log-transformed plasma levels of sIL-2R.

### Antibodies Used for Flow Cytometry

We designed two flow cytometry panels using multiple fluorochrome-conjugated antibodies. Panel 1 includes 14 markers (14 colors), which can discriminate the main mononuclear immune cell types (B cells, T cells, NK cells, monocytes, dendritic cells), and endothelial progenitor cells. Panel 2 includes 16 markers (15 colors) and focuses specifically on different types of T cells. Pacific orange dye (250 ng/ml; Life Technologies) was used as a live/dead marker in both panels. The monoclonal antibodies used in the two panels during the flow cytometry protocol are listed in **Supplementary Table 1**.

### Fluorescent Staining for Flow Cytometry

Before staining, cryopreserved PBMCs were rapidly thawed using a water bath set to 37°C and washed once in cold PBS (without calcium and magnesium, Lonza) containing 5% AB serum and Benzonase^®^ Nuclease (1:10,000; Merck Millipore) by centrifugation at 450 x g for 5 min at 4°C. The PBMCs were then resuspended in cold PBS and stained with pacific orange (250 ng/ml; Life Technologies) for 20 min on ice in the dark. Following live/dead staining, cells were washed once, taken up in cold FACS-buffer (PBS containing 0.5% BSA) and incubated with 2 µl Fc receptor block (Miltenyi Biotec) per 1 x 10^6^ cells for 10 min on ice. Cells were then subdivided into two parts and incubated for 30 min on ice in the dark with the respective antibody staining panel. The samples were subsequently washed once and re-suspended in FACS-buffer prior to analysis at the flow cytometer.

### Flow Cytometry Analysis

Samples were acquired on a LSRI Fortessa flow cytometer (BD Biosciences) with BD FACSDiva™ Software (BD Biosciences) at the Bergen Flow Cytometry Core Facility, University of Bergen, Norway. The flow cytometer was equipped with 407, 488, 561, and 635 nm lasers, and emission filters for PerCP-Cy5.5 (Long Pass (LP): 685, Band Pass (BP): 695/40), Alexa Fluor 488 (LP: 505, BP: 530/30), PE-Cy7 (LP: 750, BP: 780/60), PE (LP: -, BP: 582/15), APC (LP: -, BP: 670/14), Pacific blue (LP: -, BP: 450/50), Pacific orange (LP: 570, BP: 585/42), and BV786 (LP: 750, BP: 780/60). The cytometer was routinely calibrated with BD cytometer setup and tracking beads (BD Biosciences). An average of 138,635 (panel 1) and 122,287 (panel 2) events were recorded in the intact single cell gate and mean percentage of live cells was 98 and 96% for panels 1 and 2, respectively. Flow cytometry data was analyzed in FlowJo™ 10 (Tree Star). Compensation beads (eBioscience) stained with the respective antibody were used as controls to calculate the compensation matrix. The representative gating strategies for both panels are shown in **Supplementary Figures 1** and **2** and were validated with the unsupervised gating method using the tSNE algorithm (**Supplementary Figures 3B, C**). For accurate gating, fluorescence minus one (FMO) controls were run regularly for both panels.

### Statistical Analysis

sIL-2R was log2 transformed prior to analysis. Values above the ordinary range were removed by biological consideration and literature review. The Mann-Whitney U test was used in the comparison between the complication groups in the analyses of plasma cytokines. To evaluate the association between two variables we used the Pearson correlation formula. In flow cytometry analysis, multiple linear regression was applied and adjusted for the age and sex covariates. Differences were considered statistically significant when p <0.05. The study was of exploratory nature and hence no correction was made for multiple comparisons. Comparisons between patient groups, correlations and the production of associated graphs were done using R Studio (Version 1.1.456). Figures were arranged in Adobe Illustrator CS6.

## Results

### Elevated sIL-2R in T1D Individuals

Baseline characteristics of DIALONG study participants are given in **Table 1**. There were 26 T1D individuals with vascular complications (progressor, P), of whom 10 had CKD, 11 had CVD, and all apart from one had PDR. As the matching groups we included 25 T1D individuals without any vascular complications (non-progressors, NP) and 28 healthy controls. In brief, progressors had significantly higher BMI and slightly elevated HbA1c. The groups were balanced regarding age, sex, and eGFR.

**Table 1:** Clinical characteristics of the DIALONG study participants.

Cohort	Healthy control	NP	Progressors	p-value
N	28	25	26	
Age (years)	62.2 ± 6.3	63.1 ± 6.5	62.2 ± 6.5	ns
BMI (kg/m^3^)	26.6 ± 4.2^a^	25.1 ± 3.3	27.3 ± 3.9	3.66 × 10^-2^
Diabetes duration (years)	NA	50.5 ± 3.4	51.3 ± 5.1	ns
Age at diagnosis (years)	NA	12.6 ± 5.6	10.8 ± 6.5	ns
Sex (% female)	57%	48%	54%	ns
HbA1c (%)	5.5 ± 0.2	7.3 ± 0.8	7.6 ± 0.8	ns
GAD AA positive (%)	7%	29%^b^	32%^c^	ns
IA-2 AA positive (%)	4%	8%^b^	16%^c^	ns
Insulin AA positive (%)	0%	71%^b^	68%^c^	ns
ZnT8 AA positive (%)	0%	4%^b^	8%^c^	ns
AA positive (%)	7%	75%^b^	80%^c^	ns
eGFR (ml/min/1.73 m^3^)	83.4 ± 16.4	85.4 ± 15.1	78.5 ± 26.1	ns
C-peptide (nmol/L)	718.3 ± 225.3	undetectable	undetectable	ns
CRP (mg/L)	1.8 ± 2.3^d^	3.3 ± 6.1^e^	3.0 ± 3.3^e^	ns
Statins	6 (21%)	9 (36%)	17 (65%)	3.87 × 10^-2^
Beta-blocker	1 (4%)	2 (8%)	10 (38%)	1.16 × 10^-2^
ACEi/ARB	6 (21%)	7 (28%)	19 (73%)	1.49 × 10^-3^
Antiplatelet agent	6 (21%)	1 (4%)	14 (54%)	1.16 × 10^-4^
Loop diuretics	0 (0%)	1 (4%)	7 (27%)	2.69 × 10^-2^
PDR/CKD/CVD (n)	NA	NA	25/10/11	

Values for continuous variables are presented as mean ± SD. P-values were calculated between NPs and progressors by Mann–Whitney U test. NP, non-progressor; BMI, body mass index; HbA1c, haemoglobin A1c; GAD, glutamic acid decarboxylase; AA, autoantibody; IA2, islet cell antigen-2; ZnT8, zink transporter 8; eGFR, estimated glomerular filtration rate; C-peptide, connecting peptide; CRP, C-reactive protein; ACEi, angiotensin-converting enzyme; ARB, angiotensin receptor blocker; PDR, proliferative diabetic retinopathy; CKD, chronic kidney disease; CVD, cardiovascular disease.

^a^n = 27, ^b^n = 24; ^c^n = 25, ^d^n = 21, ^e^n = 20.

**Figure 1:**
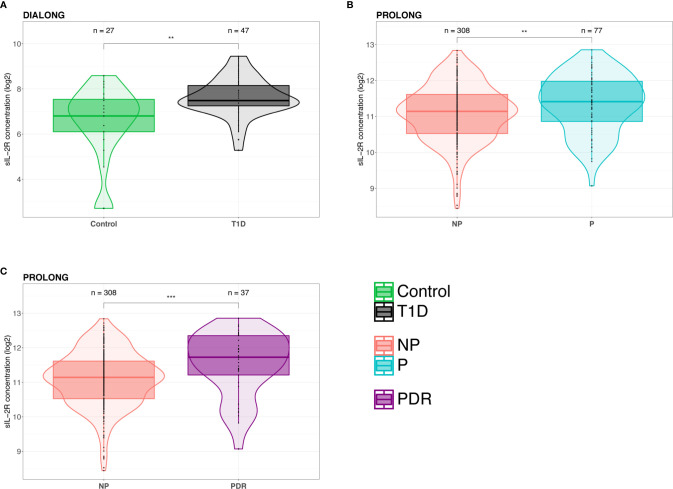
Elevated plasma levels of sIL-2R in patients with type 1 diabetes (T1D). **(A)** Patients with T1D from the DIALONG cohort had significantly elevated sIL-2R plasma levels compared to healthy controls. **(B)** In PROLONG, sIL-2R plasma levels were significantly increased in T1D with vascular complications (progressors, P) compared to T1D patients without complications (non-progressors, NP). **(C)** PROLONG progressors with proliferative diabetic retinopathy (PDR) showed significantly higher sIL-2R plasma levels compared to progressors with other vascular complications. (The Mann-Whitney U test was used in the comparison between the different groups. **p < 0.01 and ***p < 0.001).

There was a significant increase of sIL-2R (p = 0.0011) in T1D as compared to healthy individuals (**Figure 1A**). The increase was gradual in relation to vascular complication status, being highest in the progressor group (Control vs. NP: p = 0.014; Control vs. P: p = 0.0021; NP vs. P: p = 0.47) (**Supplementary Figure 4A**). None of the other analyzed cytokines showed significant differences between the T1D groups in relation to their complication status (**Supplementary Figure 5A**). An overview over the detection rate for each investigated cytokine is provided in **Supplementary Figure 5B**.

To investigate complication status further, we stratified progressors into those with CKD, CVD, or PDR. These analyses revealed that progressors with CVD had significantly elevated sIL-2R plasma levels (p = 0.029) as compared to NPs (**Supplementary Figure 4B**). Plasma sIL-2R was slightly increased in progressors with CKD (p = 0.19) as compared to NPs (**Supplementary Figure 4C**), and sIL-2R correlated negatively with eGFR (T1D: R = -0.42, p = 0.0037) (**Supplementary Figure 4D**). Adjusting for eGFR did not change the observed result with CKD (p = 0.35). Plasma sIL-2R was elevated in progressors with PDR compared to NPs (p = 0.36) (**Supplementary Figure 4E**). Monocyte chemotactic protein-1 (CCL2, alternative: MCP-1) plasma levels were significantly higher in progressors with CKD (p = 0.021) and CVD (p = 0.013) compared to NPs (**Supplementary Figures 4F, G**). CCL2 was slightly elevated in progressors with PDR compared to NPs (p = 0.09) (**Supplementary Figure 4H**).

### Elevated sIL-2R in T1D Individuals with Vascular Complications

In order to confirm our cytokine findings in DIALONG in a larger and independent cohort, we measured 4 nominally associated cytokines (sIL-2R, CCL2, CCL11, IFN-α) in plasma from PROLONG patients with T1D with and without complications (n = 394). Clinical characteristics for this cohort are summarized in **Table 2**. We included 81 patients with T1D with vascular complications (progressors, P), of whom 40 had PDR, 58 had CKD, and on 2 we had information on CVD. Those individuals were compared to 313 T1D individuals without any vascular complications (non-progressors, NP). Progressors were significantly younger in age, displayed significantly higher BMI and HbA1c and lower eGFR and diabetes duration compared to NPs. The groups were balanced regarding sex.

**Table 2:** Clinical characteristics of the PROLONG participants.

Cohort	Cytokine assay			Flow cytometry		
	NP	Progressors	p-value	NP	Progressors	p-value
n	313	81		44	17	
Age (yrs.)	58.1 ± 10.6	44.6 ± 13.7^a^	1.08 × 10^-13^	50.6 ± 7.2	50.8 ± 15.5	ns
BMI (kg/m^3^)	24.8 ± 3.7^b^	26.4 ± 4.7	3.94 × 10^-3^	25.3 ± 4.2	25.3 ± 4.7	ns
Diabetes duration (years)	40.6 ± 8.6	22.4 ± 8.3	<2.2 × 10^-16^	37.7 ± 5.1	29.9 ± 13.4	9.49 × 10^-3^
Age at diagnosis (years)	17.5 ± 9.9	22.0 ± 14.4^a^	ns	12.9 ± 6.0	20.9 ± 13.2	4.63 × 10^-2^
Sex (% female)	58%	53%	ns	59%	53%	ns
HbA1c (%)	7.6 ± 0.9	8.9 ± 1.5	3.33 × 10^-14^	7.4 ± 0.9	8.5 ± 0.8	6.35 × 10^-5^
GAD AA positive (%)	50%^c^	68%	8.15 × 10^-3^	50%^d^	90%^e^	2.41 × 10^-2^
eGFR (ml/min/1.73 m^3^)	90.2 ± 15.5	97.4 ± 30.8^f^	3.66 × 10^-3^	94.6 ± 16.6	93.3 ± 29.7	ns
C-peptide (nmol/L)	0.03 ± 0.07^g^	0.03 ± 0.05^h^	ns	0.01 ± 0.02^i^	0.02 ± 0.01^j^	3.37 × 10^-2^
PDR/CKD/CVD (n)	NA	40/58/2		NA	10/12/0	

Values for continuous variables are presented as mean ± SD. P-values were calculated by Mann–Whitney U test. NP, non-progressor; BMI, body mass index; HbA1c, haemoglobin A1c; GAD, glutamic acid decarboxylase; AA, autoantibody; eGFR, estimated glomerular filtration rate; C-peptide, connecting peptide; PDR, proliferative diabetic retinopathy; CKD, chronic kidney disease; CVD, cardiovascular disease.

^a^n = 77; ^b^n = 308; ^c^n = 303; ^d^n = 40; ^e^n = 10; ^f^n = 76; ^g^n = 296; ^h^n = 68; ^i^n = 35; ^j^n = 7.

Three cytokines were detected in 100% of samples (sIL-2R, CCL2, CCL11), while IFN-α was only detected <16% of samples (**Supplementary Figure 6B**). We observed a significant increase of sIL-2R (p = 0.0064) in progressors compared to NPs (**Figure 1B**). This observed difference was even more pronounced (p = 0.00084) when comparing NPs with progressors with PDR (**Figure 1C**). Additionally, sIL-2R was slightly increased in patients with CKD (p = 0.077) compared to NPs (**Supplementary Figure 6C**), and sIL-2R correlated negatively with eGFR (R = -0.12, p = 0.025) (**Supplementary Figure 6D**). Adjusting for eGFR using linear regression, resulted in a significant increase in sIL-2R between progressors with CKD and NPs (p = 0.041). Comparisons for CVD could not be made due to the small sample size of progressors with information on CVD (n = 2). As observed in the DIALONG cohort, there was no significant difference between the complication groups in CCL2 (p = 0.46), CCL11 (p = 0.25), and IFN- α (p = 0.40) (**Supplementary Figure 6A**). In PROLONG, CCL2 was not elevated in progressors with CKD (p = 0.39) (**Supplementary Figure 6E**). Progressors with PDR and NPs showed similar levels of CCL2 (p = 0.97) (**Supplementary Figure 6F**).

### Association of sIL-2R Levels With IL2RA and PTPN2 Gene Variants

To identify associations between genetic variants in *IL2RA* and plasma levels of sIL-2R, we used linear regression adjusting for sex, age, and group. Plasma levels of sIL-2R were significantly associated with 68 SNPs in the *IL2RA* gene (**Table 3**), with rs12722489 showing the statistically strongest association (p = 5,19 × 10^-7^). Two of our identified SNPs are located in exon 8, namely rs12722606 and rs12722605. The majority of the associated SNPs (n = 51) are located in the large intron 1 area of *IL2RA*.

**Table 3:** Imputed *IL2RA* genotypes and their significant associations with sIL-2R in plasma.

Chr	SNP	bp*	Intron/Exon	A1	n	Beta (95% CI)	p-Value
10	rs12722489	6102012	Intron 1	T	330	−0.28 (−0.39, −0.17)	5.19 × 10^−7^
10	rs12722517	6081040	Intron 1	C	330	−0.24 (−0.34, −0.14)	2.23 × 10^−6^
10	rs9663421	6055604	Intron 7	T	330	0.25 (0.15, 0.35)	3.17 × 10^−6^
10	rs12722553	6071284	Intron 1	A	330	−0.26 (−0.37, −0.15)	3.30 × 10^−6^
10	rs12722551	6071379	Intron 1	T	330	−0.26 (−0.37, −0.15)	3.30 × 10^−6^
10	rs791593	6083292	Intron 1	G	330	−0.23 (−0.33, −0.14)	4.22 × 10^−6^
10	rs2104286	6099045	Intron 1	C	330	−0.24 (−0.33, −0.14)	4.63 × 10^−6^
10	rs41294713	6080354	Intron 1	C	330	−0.25 (−0.36, −0.15)	5.23 × 10^−6^
10	rs12722515	6081230	Intron 1	A	330	−0.25 (−0.36, −0.15)	5.23 × 10^−6^
10	rs791590	6090322	Intron 1	T	330	−0.25 (−0.36, −0.15)	5.23 × 10^−6^
10	rs2246031	6092210	Intron 1	T	330	−0.25 (−0.36, −0.15)	5.23 × 10^−6^
10	rs7078614	6075831	Intron 1	T	330	−0.21 (−0.30, −0.12)	5.50 × 10^−6^
10	rs7920946	6074634	Intron 1	C	330	−0.22 (−0.31, −0.13)	5.53 × 10^−6^
10	rs4625363	6072504	Intron 1	G	330	−0.25 (−0.36, −0.15)	5.54 × 10^−6^
10	rs12722527	6077328	Intron 1	T	330	−0.25 (−0.36, −0.15)	5.54 × 10^−6^
10	rs12722523	6078390	Intron 1	A	330	−0.25 (−0.36, −0.15)	5.54 × 10^−6^
10	rs12722561	6069893	Intron 1	T	330	−0.25 (−0.36, −0.15)	6.22 × 10^−6^
10	rs12722559	6070273	Intron 1	A	330	−0.25 (−0.36, −0.15)	6.22 × 10^−6^
10	rs12722606	6053133	Exon 8	A	330	0.25 (0.14, 0.36)	6.98 × 10^−6^
10	rs11256335	6055222	Intron 7	A	330	0.25 (0.14, 0.36)	6.98 × 10^−6^
10	rs12722605	6053163	Exon 8	A	330	0.29 (0.17, 0.42)	7.27 × 10^−6^
10	rs12722497	6095928	Intron 1	A	330	0.35 (0.20, 0.50)	1.22 × 10^−5^
10	rs11256464	6082558	Intron 1	T	330	0.32 (0.18, 0.47)	2.55 × 10^−5^
10	rs11597237	6079017	Intron 1	T	330	−0.23 (−0.33, −0.12)	3.07 × 10^−5^
10	rs11256416	6075359	Intron 1	T	330	−0.21 (−0.31, −0.11)	3.67 × 10^−5^
10	rs7910961	6077796	Intron 1	T	330	0.20 (0.11, 0.30)	4.04 × 10^−5^
10	rs4747837	6058735	Intron 7	G	330	−0.23 (−0.34, −0.12)	4.34 × 10^−5^
10	rs7900385	6062748	Intron 4	A	330	−0.23 (−0.34, −0.12)	4.34 × 10^−5^
10	rs12722588	6060433	Intron 6	T	330	−0.22 (−0.33, −0.11)	5.83 × 10^−5^
10	rs12722587	6060630	Intron 6	T	330	−0.22 (−0.33, −0.11)	5.83 × 10^−5^
10	rs7093069	6063319	Intron 4	T	330	−0.22 (−0.33, −0.11)	5.83 × 10^−5^
10	rs11816044	6074082	Intron 1	A	330	0.20 (0.10, 0.30)	8.91 × 10^−5^
10	rs7100984	6078539	Intron 1	A	330	0.20 (0.10, 0.30)	9.07 × 10^−5^
10	rs12722574	6066462	Intron 2	A	330	−0.20 (−0.29, −0.10)	9.63 × 10^−5^
10	rs4749894	6058323	Intron 7	G	330	0.22 (0.11, 0.33)	1.00 × 10^−4^
10	rs4749924	6082396	Intron 1	C	330	0.20 (0.10, 0.29)	1.00 × 10^−4^
10	rs6602398	6082953	Intron 1	T	330	0.20 (0.10, 0.29)	1.00 × 10^−4^
10	rs7900744	6065611	Intron 3	G	330	−0.20 (−0.29, −0.10)	1.19 × 10^−4^
10	rs791588	6089342	Intron 1	G	330	0.15 (0.06, 0.24)	7.71 × 10^−4^
10	rs11256342	6057231	Intron 7	G	330	0.17 (.07, 0.26)	8.37 × 10^−4^
10	rs12253981	6092346	Intron 1	G	330	0.16 (0.07, 0.25)	8.84 × 10^−4^
10	rs2025345	6067688	Intron 2	G	330	0.16 (0.07, 0.26)	9.75 × 10^−4^
10	rs12358961	6066195	Intron 3	A	330	0.16 (0.07, 0.26)	1.03 × 10^−3^
10	rs1924138	6096158	Intron 1	A	330	0.16 (0.06, 0.25)	1.06 × 10^−3^
10	rs11256497	6087794	Intron 1	A	330	0.15 (0.06, 0.25)	1.36 × 10^−3^
10	rs10795752	6072354	Intron 1	T	330	0.15 (0.06, 0.25)	1.51 × 10^−3^
10	rs2245675	6095577	Intron 1	A	330	0.16 (0.06, 0.25)	1.55 × 10^−3^
10	rs2256852	6096923	Intron 1	A	330	0.16 (0.06, 0.25)	1.55 × 10^−3^
10	rs791587	6088699	Intron 1	A	330	0.14 (0.05, 0.23)	1.62 × 10^−3^
10	rs12251836	6091281	Intron 1	A	330	0.15 (0.06, 0.24)	1.65 × 10^−3^
10	rs6602368	6062915	Intron 4	C	330	0.15 (0.06, 0.24)	1.67 × 10^−3^
10	rs2476491	6095410	Intron 1	T	330	0.16 (0.06, 0.26)	1.74 × 10^−3^
10	rs4749926	6085312	Intron 1	A	330	0.15 (0.05, 0.24)	2.60 × 10^−3^
10	rs11256457	6080794	Intron 1	G	330	0.15 (0.05, 0.24)	2.72 × 10^−3^
10	rs10905641	6072293	Intron 1	C	330	0.14 (0.05, 0.23)	3.41 × 10^−3^
10	rs6602379	6073374	Intron 1	G	330	0.14 (0.05, 0.24)	3.51 × 10^−3^
10	rs809356	6091148	Intron 1	C	330	0.13 (0.04, 0.22)	3.60 × 10^−3^
10	rs2256774	6097165	Intron 1	C	330	0.14 (0.05, 0.23)	3.87 × 10^−3^
10	rs1323657	6072427	Intron 1	A	330	0.13 (0.04, 0.23)	3.95 × 10^−3^
10	rs7072398	6079846	Intron 1	A	330	0.13 (0.04. 0.22)	5.33 × 10^−3^
10	rs10795763	6096199	Intron 1	G	330	0.13 (0.04. 0.22)	5.34 × 10^−3^
10	rs7917726	6096600	Intron 1	G	330	0.13 (0.04. 0.22)	5.34 × 10^−3^
10	rs706779	6098824	Intron 1	C	330	0.12 (0.03. 0.21)	7.97 × 10^−3^
10	rs10905656	6086093	Intron 1	A	330	0.11 (0.02. 0.20)	1.51 × 10^−2^
10	rs3793713	6059704	Intron 7	G	330	0.11 (0.01. 0.20)	2.56 × 10^−2^
10	rs4749920	6071453	Intron 1	C	330	0.11 (0.01. 0.21)	3.73 × 10^−2^
10	rs4749921	6071654	Intron 1	C	330	0.11 (0.01. 0.21)	3.73 × 10^−2^
10	rs4747845	6074441	Intron 1	A	330	0.11 (0.01. 0.21)	3.73 × 10^−2^

*SNP positions according to the Genome Reference Consortium Human Build 37 (GRch37).

Linear regression model: p-value = adjusted for age, sex and complication group. Chr, chromosome; SNP, single nucleotide polymorphism; bp, base pair; A1, minor allele; CI, confidence interval.

Furthermore, sIL-2R levels were significantly associated with 53 intronic SNPs in the *PTPN2* gene region (**Table 4**). The variant rs12971201 showed the strongest association with plasma sIL-2R (p = 1.09 × 10^-3^). When we conditioned this analysis for the T1D-associated SNP rs2104286 in *IL2RA*, we could still identify 42 *PTPN2* variants to be independently associated with sIL-2R (**Table 4**). Here rs592390 had the strongest association with plasma sIL-2R (p = 2.16 × 10^-3^).

**Table 4:** Imputed *PTPN2* genotypes and their significant associations with sIL-2R in plasma.

Chr	SNP	bp*	Intron/Exon	A1	n	Beta (95% CI)	p-Value_a_	p-Value_b_
18	rs12971201	12830538	Intron 4	A	330	0.16 (0.07, 0.26)	1.09 × 10^−3^	3.01 × 10^−3^
18	rs2542162	12820900	Intron 5	T	330	0.16 (0.06, 0.26)	1.13 × 10^−3^	2.91 × 10^−3^
18	rs2847281	12821593	Intron 5	G	330	0.16 (0.06, 0.26)	1.13 × 10^−3^	2.91 × 10^−3^
18	rs2852151	12841176	Intron 2	A	330	0.16 (0.06, 0.26)	1.16 × 10^−3^	3.38 × 10^−3^
18	rs3826557	12843263	Intron 2	T	330	0.16 (0.06, 0.26)	1.16 × 10^−3^	3.38 × 10^−3^
18	rs674222	12848349	Intron 2	C	330	0.16 (0.06, 0.26)	1.16 × 10^−3^	3.38 × 10^−3^
18	rs2847273	12856908	Intron 2	C	330	0.16 (0.06, 0.26)	1.16 × 10^−3^	3.38 × 10^−3^
18	rs641085	12824930	Intron 5	T	330	0.15 (0.06, 0.24)	1.61 × 10^−3^	2.22 × 10^−3^
18	rs592390	12822314	Intron 5	C	330	0.15 (0.06, 0.24)	1.67 × 10^−3^	2.16 × 10^−3^
18	rs12957037	12829065	Intron 4	G	330	0.15 (0.06, 0.24)	1.67 × 10^−3^	2.16 × 10^−3^
18	rs588447	12832842	Intron 3	C	330	0.15 (0.06, 0.24)	1.69 × 10^−3^	2.48 × 10^−3^
18	rs8087237	12834359	Intron 3	A	330	0.15 (0.06, 0.24)	1.69 × 10^−3^	2.48 × 10^−3^
18	rs478582	12835976	Intron 3	C	330	0.15 (0.06, 0.24)	1.69 × 10^−3^	2.48 × 10^−3^
18	rs559406	12857002	Intron 2	G	330	−0.14 (−0.24, −0.05)	1.94 × 10^−3^	2.44 × 10^−3^
18	rs960550	12827697	Intron 4	T	330	0.15 (0.06, 0.25)	2.08 × 10^−3^	6.02 × 10^−3^
18	rs4797709	12882359	Intron 1	C	330	0.15 (0.05, 0.24)	2.43 × 10^−3^	6.64 × 10^−3^
18	rs2292759	12884343	upstream	A	330	0.15 (0.05, 0.24)	2.43 × 10^−3^	8.09 × 10^−3^
18	rs2542157	12787247	Intron 10	G	330	0.14 (0.04, 0.23)	5.57 × 10^−3^	4.98 × 10^−3^
18	rs2847291	12808713	Intron 8	A	330	0.14 (0.04, 0.24)	6.86 × 10^−3^	1.43 × 10^−2^
18	rs11663472	12810471	Intron 8	A	330	0.14 (0.04, 0.24)	6.86 × 10^−3^	1.43 × 10^−2^
18	rs2847286	12817815	Intron 6	G	330	0.14 (0.04, 0.24)	6.86 × 10^−3^	1.43 × 10^−2^
18	rs2847285	12818224	Intron 6	A	330	0.14 (0.04, 0.24)	6.86 × 10^−3^	1.43 × 10^−2^
18	rs45456495	12792228	Intron 10	T	330	0.13 (0.03, 0.24)	9.11 × 10^−3^	1.77 × 10^−2^
18	rs2542167	12795849	Intron 9	T	330	0.13 (0.03, 0.24)	9.11 × 10^−3^	1.77 × 10^−2^
18	rs2847298	12800120	Intron 9	G	330	0.13 (0.03, 0.24)	9.11 × 10^−3^	1.77 × 10^−2^
18	rs2542160	12789246	Intron 10	C	330	0.13 (0.03, 0.23)	1.06 × 10^−2^	1.94 × 10^−2^
18	rs2847299	12801337	Intron 9	A	330	0.14 (0.03, 0.24)	1.10 × 10^−2^	3.06 × 10^−2^
18	rs7227207	12819616	Intron 5	T	330	−0.13 (−0.23, −0.03)	1.15 × 10^−2^	2.13 × 10^−2^
18	rs72872125	12876915	Intron 1	T	330	0.19 (0.04, 0.34)	1.24 × 10^−2^	1.72 × 10^−2^
18	rs60474474	12792736	Intron 10	T	330	−0.14 (−0.25, −0.03)	1.68 × 10^−2^	2.03 × 10^−2^
18	rs45450798	12792940	Intron 10	G	330	−0.14 (−0.25, −0.03)	1.68 × 10^−2^	2.03 × 10^−2^
18	rs60751993	12795420	Intron 9	A	330	−0.14 (−0.25, −0.03)	1.68 × 10^−2^	2.03 × 10^−2^
18	rs60735058	12795470	Intron 9	A	330	−0.14 (−0.25, −0.03)	1.68 × 10^−2^	2.03 × 10^−2^
18	rs8096138	12808140	Intron 8	G	330	−0.14 (−0.25, −0.03)	1.68 × 10^−2^	2.03 × 10^−2^
18	rs1893217	12809340	Intron 8	G	330	−0.14 (−0.25, −0.03)	1.68 × 10^−2^	2.03 × 10^−2^
18	rs11663253	12789556	Intron 10	G	330	−0.13 (−0.25, −0.02)	1.84 × 10^−2^	2.12 × 10^−2^
18	rs10502416	12822702	Intron 5	T	330	−0.13 (−0.24, −0.02)	2.01 × 10^−2^	2.14 × 10^−2^
18	rs78637414	12826836	Intron 4	A	330	−0.13 (−0.24, −0.02)	2.01 × 10^−2^	2.14 × 10^−2^
18	rs62097820	12834649	Intron 3	T	330	−0.13 (−0.24, −0.02)	2.01 × 10^−2^	2.14 × 10^−2^
18	rs8096327	12887750	Upstream	G	330	−0.10 (−0.19, −0.01)	2.91 × 10^−2^	3.71 × 10^−2^
18	rs3737361	12831324	Intron 3	C	330	−0.11 (−0.22, −0.01)	3.07 × 10^−2^	5.45 × 10^−2^
18	rs16939910	12837993	Intron 2	A	330	−0.11 (−0.22, −0.01)	3.07 × 10^−2^	5.45 × 10^−2^
18	rs3786158	12843275	Intron 2	A	330	−0.11 (−0.22, −0.01)	3.07 × 10^−2^	5.45 × 10^−2^
18	rs11080605	12847329	Intron 2	C	330	−0.11 (−0.22, −0.01)	3.07 × 10^−2^	5.45 × 10^−2^
18	rs62097858	12862581	Intron 1	A	330	−0.11 (−0.22, −0.01)	3.07 × 10^−2^	5.45 × 10^−−2^
18	rs8091720	12865186	Intron 1	T	330	−0.11 (−0.22, −0.01)	3.07 × 10^−2^	5.45 × 10^−2^
18	rs7244152	12854294	Intron 2	C	330	−0.11 (−0.22, −0.01)	3.23 × 10^−2^	5.44 × 10^−2^
18	rs11080606	12867969	Intron 1	C	330	−0.11 (−0.22, −0.01)	3.23 × 10^−2^	5.44 × 10^−2^
18	rs7242788	12820330	Intron 5	A	330	−0.11 (−0.22, −0.01)	3.30 × 10^−2^	5.79 × 10^−2^
18	rs12959799	12900695	Upstream	G	330	0.11 (0.01, 0.21)	4.01 × 10^−2^	5.41 × 10^−2^
18	rs80262450	12818922	Intron 6	A	330	−0.13 (−0.25, −0.01)	4.13 × 10^−2^	3.45 × 10^−2^
18	rs56946650	12916943	Upstream	T	330	−0.11 (−0.22, −0.00)	4.16 × 10^−2^	6.45 × 10^−2^
18	rs2847282	12819820	Intron 5	G	330	−0.09 (−0.19, −0.00)	4.79 × 10^−2^	6.62 × 10^−2^

*SNP positions according to the Genome Reference Consortium Human Build 37 (GRch37)

Linear regression models: p-value_a_ = adjusted for age, sex and complication group; p-value_b_ = adjusted for age, sex, complication group and rs2104286. Chr, chromosome; SNP, single nucleotide polymorphism; bp, base pair; A1, minor allele; CI, confidence interval.

### Cell-Surface Marker Analysis on PBMC of T1D Individuals

Our flow cytometry panels enabled us to investigate a range of different cell populations, which are summarized in **Supplementary Figure 3A**. The applied gating strategies are provided in **Supplementary Figure 1** and **2**. Baseline characteristics for this subset of PROLONG patients are provided in **Table 2**. In total, we performed flow cytometry analysis on 61 T1D samples, of which 17 were from progressors. The groups were balanced regarding age and sex.

**Figure 2:**
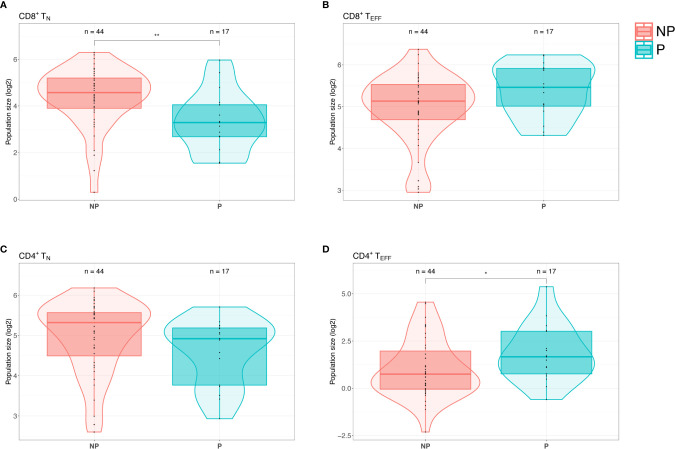
Patients with type 1 diabetes (T1D) with complications display a shift from naïve T cells to effector T cells. **(A)** Flow cytometry screening of PBMCs from PROLONG patients revealed a significant decrease of CD8^+^ naïve T (T_N_) cells (CD3^+^CD4^+^CD45RA^+^CCR7^+^CCR5^-^) in progressors (P) compared to non-progressors (NP). **(B)** Progressors displayed elevated CD8^+^ effector T (T_EFF_) cells (CD3^+^CD4^+^CD45RA^+^CCR7^-^) simultaneously (p = ns). **(C)** CD4^+^ T_N_ cells were also declined in patients with T1D with complications as compared to NPs (p = ns). **(D)** In progressors CD4^+^ T_EFF_ cells were significantly elevated compared to NPs. (For the comparison between the different groups, multiple linear regression was applied and adjusted for the age and sex covariates. *p < 0.05 and **p < 0.01).

We identified a significant decrease of CD8^+^ naïve T cells (CD3^+^CD8^+^CD45RA^+^CCR7^+^CCR5^-^) (p = 0.0046) and increase of CD8^+^ effector T cells (CD3^+^CD8^+^CD45RA^+^CCR7^-^) (p = 0.070) in progressors compared to NPs (**Figures 2A, B**). Furthermore, progressors had significantly increased CD4^+^ effector T cells (CD3^+^CD4^+^CD45RA^+^CCR7^-^) (p = 0.045) and decreased CD4^+^ naïve T cells (CD3^+^CD4^+^CD45RA^+^CCR7^+^CCR5^-^) (p = 0.14) compared to NPs (**Figures 2C, D**). To summarize, we observed a shift from naïve to effector T cells (CD4^+^ and CD8^+^) in PBMCs from T1D patients with vascular complications compared to those without.

Since we observed significant differences in plasma sIL-2R in the two PROLONG groups, we also investigated cell surface expression of interleukin-2 receptor alpha (CD25) on PBMCs. Remarkably, we did not see differences between NPs and progressors in neither CD25^+^ T cells (p = 0.84) nor Tregs (CD3^+^CD4^+^CD25^++^CD127^-^) (p = 0.27) (**Supplementary Figures 7A, B**).

## Discussion

The present study revealed that plasma sIL-2R levels are reproducibly elevated in individuals with long-term T1D with severe vascular complications as compared to those who remained free from to vascular complications despite more than 30 years of diabetes duration. Further, plasma levels of sIL-2R were associated with SNPs in the *IL2RA* and *PTPN2* gene regions, which might suggest underlying genetic determinants and possibly biological causal inference. Finally, our results are in agreement with published studies confirming an increase of circulating sIL-2R in patients with T1D when compared to healthy controls, which might further emphasize that immune factors contributing to diabetes pathogenesis might early on govern progression to vascular complications (12, 23).

The biological function of sIL-2R is not yet completely understood, but there is evidence that it reflects an imbalance in Treg and effector T cell (T_EFF_) activity (14, 24). It has been suggested that there is a reduced immunosuppressive function of Tregs due to impaired IL-2 signalling in T1D (24–28), a defect which may subsequently lead to a more aggressive immune destruction of pancreatic β cells by T_EFF_ (12, 28). In addition, defects in the intracellular IL-2 pathways and a decreased regulatory function have recently been reported in patients with type 2 diabetes (T2D) (29). In many autoimmune diseases, such as multiple sclerosis (MS) (30), rheumatoid arthritis (31), primary Sjögren’s syndrome (32), scleroderma (33), and inflammatory myopathies (34), but also in various cancers (31), sIL-2R has been proposed to be a biomarker for disease activity.

Patients with T1D develop the disease at an early age and a large proportion of them will progress to devastating vascular complications representing a major problem because the tools for monitoring when and how disease deteriorates are not available (4). Diabetes retinopathy is the most common vascular complication of diabetes (35) and the proliferative form of diabetic retinopathy (PDR) is the leading cause of vision loss in adults (36). Previous studies show that several inflammatory cytokines are involved in the pathogenesis and progression of PDR, including sIL-2R (19), however, not all of the results have been replicated. In the present study, both PROLONG and DIALONG progressors with PDR had higher plasma levels of sIL-2R compared to NPs, supporting the notion that sIL-2R could emerge as a contributing player not only in the pathogenesis of T1D but also in disease progression.

An additional evidence in the present study to biological importance of sIL-2R were our findings that 68 *IL2RA* SNPs are associated with sIL-2R plasma levels in PROLONG patients with T1D and correlated with the elevated sIL-2R levels observed in progressors accordingly. *IL2RA* rs12722489 showed the strongest association with sIL-2R plasma levels in patients with T1D and is located in the large intron 1. This particular SNP has been identified as a risk factor for MS in several studies (37–39). However, a secondary association was suggested due to the nearby location of rs2104286 (Linkage disequilibrium D’ = 1, R^2^ = 0.58), which is a well-recognized T1D risk factor (11, 37) and also significantly associated with plasma sIL-2R in our dataset. Interestingly, one of our identified SNPs, rs2256774, was associated with higher levels of Rubella antibodies (40), and Rubella viral infections have been associated with the onset of T1D (41, 42). Additionally, multiple *IL2RA* variants have been shown to correlate with sIL-2R levels in T1D (10) and MS (37) and *IL2RA* gene variants are associated with susceptibility of T1D (10, 11, 43). Further, functional studies support these results by suggesting that specific *IL2RA* variants cause defects in immune homeostasis due to impaired IL-2 signalling in Tregs (25, 27, 44).

Interestingly, seventeen of our identified SNPs are positioned within the first 15 kb of the first intron of the *IL2RA* gene, which has been described as a super-enhancer region due to a cluster of histone 3 lysine 27 acetylation (H3K27ac) elements in this area (45). Notably, many of the *IL2RA* SNPs related to autoimmune diseases fall into this region and affect transcription factor binding and enhancer activity (46). Several of our identified SNPs have been reported to be associated with DNA methylation at the *IL2RA* promoter locus, particularly rs6602398 and rs4749926 (47). Despite having detected a number of *IL2RA* SNPs to be associated with sIL-2R, it is challenging to conclude the direct effect of those mostly intronic SNPs on the expression of IL-2R itself and in-depth research is scarce. However, it has been reported that one of our identified SNPs, rs12251836, is associated with *IL2RA* expression on acutely triggered T_EFF_, but not in Tregs (48), suggesting a likely biological causal inference of IL-2R gene locus in T1D.

Another exciting and supporting genetic observation included 53 *PTPN2* SNPs to be significantly associated with sIL-2R plasma levels, importantly 42 of which were associations independent of *IL2RA* variant rs2104286. Our most significant SNP in *PTPN2* is rs12971201, which has previously been associated with T1D, however a secondary association has been suggested due to rs1893217 (49). This particular variant is considered a risk variant for T1D and celiac disease (50, 51), and correlated with impaired IL-2 signalling in CD4^+^ T cells as measured by decreased phosphorylation of STAT5 (pSTAT5) but also reduced PTPN2 expression (52). One of our identified SNPs, rs2847281, was shown to significantly associate with CRP levels along many other *PTPN2* variants (53). PTPN2 is a negative regulator in the IL-2 signalling cascade and several SNPs in the *PTPN2* gene region have been linked to different autoimmune diseases including T1D, rheumatoid arthritis and Crohn’s disease (54, 55). Furthermore, different genetic variants in *PTPN2* were reported to be associated with diminished IL-2 responsiveness in naïve Tregs from patients with long-standing T1D (56). PTPN2 has been shown to modulate interferon gamma signal transduction in pancreatic β cells and regulate cytokine-induced apoptosis, which could potentially contribute to the pathogenesis of T1D (57).

As described above, elevated sIL-2R levels are likely to reflect an imbalance in Treg and T_EFF_ activity. PBMCs from PROLONG patients with T1D with vascular complications displayed a shift from naïve T cells (T_N_) to T_EFF_, however, we cannot distinguish whether this shift is the cause or the result of diabetic complications. The interpretation of these results was difficult due to the small and heterogeneous sample size in the progressors group. One can speculate that progressors have increased T_EFF_ due to impaired Treg suppression leading to a more destructive form of T1D, thereby more active course of the disease. This systemic long-term inflammation could subsequently drive the development of vascular complications by affecting tissues aside from the pancreas. Further studies testing suppression capacity of Tregs isolated from patients with T1D with and without complications are crucial to confirm this notion.

In PROLONG, we observed significantly higher levels of sIL-2R in progressors which associated with *IL2RA* SNPs, however the surface expression of IL-2R on circulating immune cells was similar between progressors and NPs. This may be confirmatory for the theory that it is not the number of cells expressing IL-2R making a difference, but the efficiency of IL-2 signalling within the cells themselves. Paradoxically, the IL-2/IL-2R signalling pathway is important in immunity and tolerance, which is further complicated by shedding of sIL-2R. How sIL-2R is involved in the pathogenesis of different diseases remains a puzzle. The high-affinity receptor for IL-2 consists of 3 protein chains, namely IL-2Rα, IL-2Rβ, and IL-2Rγ. Upon proteolytic cleavage of the ectodomains of the membrane-bound IL-2Rα and release into the extracellular space, sIL-2R retains the ability to bind IL-2 with low affinity, which can lead to different outcomes. Firstly, sIL-2R may function as a decoy-receptor reducing the bioavailability of IL-2 and favor tolerance controlled by Tregs over immunity. Tregs constitutively express the high-affinity IL-2R, which enables them to out-compete conventional T cells with the intermediate-affinity receptor (IL-2Rβ and IL-2Rγ) when less IL-2 is available, thereby boosting immune tolerance (58). This difference in affinity is exploited in clinical trials in T1D where the administration of low-dose IL-2 has shown promising effects expanding and activating Tregs (59, 60). Alternatively, the binding of sIL-2R to IL-2 can enable *in trans* presentation of IL-2 to T-cells which only express the intermediate-affinity IL-2R. Overall, increased shedding of sIL-2R and it binding to IL-2 can have opposing effects depending on the cell type affected (58).

Stratified analysis of different vascular complications revealed increased sIL-2R and CCL2 levels in DIALONG patients with CVD in comparison to patients with T1D with other diabetic complications. Elevated sIL-2R has been described as a marker for coronary artery calcification progression in both individuals with and without T1D independent of traditional CVD risk factors (20). CCL2 plays a critical role in the development of atherosclerotic plaque formation by attracting monocytes to the vessel lumen where they will differentiate into macrophages and become foam cells by the uptake of low-density lipoprotein (61). Elevated plasma levels of CCL2 have also been associated with CVD (61–63). The observed increases in sIL-2R and CCL2 were based on a small sample set in DIALONG, however, the patient characterisation for CVD was performed thoroughly using computed tomography coronary angiography, which enabled the identification of asymptomatic coronary artery disease (64). Nevertheless, we were not able to investigate this finding in PROLONG due to the limited information on CVD.

Previously it was shown in the EURODIAB study that patients with T1D with complications have increased IL-6 and TNF-α as compared to individuals without complications (9). We could neither confirm nor confound this finding due to the low detection rate of IL-6 and TNF-α (< 20%) in our study, which statistically did not allow for reliable comparisons. In general, the detection rates in our cytokine screening were considerably low, where many of the investigated biomarkers were not detected at all. This could be due to technical differences and kit quality, however all kits used were validated for plasma usage by the respective providers, we followed manufacturer’s instructions accordingly and did not experience technical issues during analysis.

Our future perspective is to unravel the role of IL-2R in the progression to diabetic complications in general, larger cohorts analyzing sIL-2R levels in other types of diabetes, such as T2D with no autoimmunity and latent autoimmune diabetes of adults, are of importance. To investigate the predictive power of sIL-2R levels in the development of diabetic complications, longitudinal studies in children and adolescents would be a great asset. Furthermore, it is of great interest to study the relationship between sIL-2R and IL-2 signalling efficacy and Treg function in patients with T1D.

In summary, we conclude that *IL2RA* and *PTPN2* gene variants may not only increase the risk of T1D, but in addition the development of diabetic complications possibly by influencing sIL-2R plasma levels and lowering T cell responsiveness. Thus, sIL-2R could potentially act as a biomarker for monitoring vascular complications in people with T1D thereby enabling early treatment and improving patient care.

## Data Availability Statement

The datasets presented in this article are not readily available because of GDPR and ethical restrictions. Requests to access the datasets should be directed to valeriya.lyssenko@uib.no.

## Ethics Statement

The studies involving human participants were reviewed and approved by PROLONG-Sweden: Regional Ethics Review Board, Department 1, Lund, Sweden, Dnr 777/2009; PROLONG-Denmark: Scientific-Ethical Committee for the Capital Region of Denmark, Hillerød, Denmark, Dnr H-2-2013-073; DIALONG: Regional Committees for Medical and Health Research Ethics (REC), South-East regional health authority, panel D, Norway, 2014/851; Data analysis of both studies at University of Bergen: REC, West regional health authority, Norway 2019/1324. The patients/participants provided their written informed consent to participate in this study.

## Author Contributions

VL, RJ, and SA conceived the study, and MK and SA designed the immunological part of the study. TM, CL, HF, TN, S-BC, GJ, LG, ME, BE, KB, and PN contributed to the study design and data collection. VL is the PI of the PROLONG study. TB is the PI of the DIALONG study. MK conducted the flow cytometric and cytokine analysis and wrote the manuscript. MK, OF, EP, BB, and SA analyzed and processed the data. All authors contributed to the article and approved the submitted version.

## Funding

Financial support was obtained from the Swedish Research Council (Dnr2015-03574, Dnr349-2006-237), the Novo Nordisk Foundation (NNF12OC1016467), the Family Erling-Person Foundation, the Steno Diabetes Center Copenhagen, the Bergen Research Foundation (BFS811294), and the University of Bergen. The DIALONG study is supported by the Oslo Diabetes Research Centre and the Norwegian Diabetics’ Centre.

## Conflict of Interest

The authors declare that the research was conducted in the absence of any commercial or financial relationships that could be construed as a potential conflict of interest. At the time when the data from Steno was collected, CLM was affiliated with Steno Diabetes Centre. During revisions and the finalization of the article, CLM have changed affiliation from Steno to Novo Nordisk as of 6-Jun-2016.
