# Supplementary material for: Increased Plasma Soluble Interleukin-2 Receptor Alpha Levels in Patients With Long-Term Type 1 Diabetes With Vascular Complications Associated With IL2RA and PTPN2 Gene Polymorphisms
Source: Front Endocrinol (Lausanne). 2020 Oct 30;11:575469. doi: 10.3389/fendo.2020.575469 (PMC7664831; doi:10.3389/fendo.2020.575469)
Supplement: Supplementary file 1 [file DataSheet_1.pdf]

Supplementary Material

Supplementary Tables

Supplementary Table 1. Listing of all applied fluorescently labelled antibodies used for both flow cytometry panels.

Marker	Conjugate	Clone	Dilution	Producer	Panel
CD1c	APC	L161	1:100	BioLegend	1
CD3	Brilliant Violet 785™	UCHT1	1:25	BioLegend	1
	Alexa Fluor® 700	UCHT1	1:100	BioLegend	2
CD4	APC-Cy™7	RPA-T4	1:250	BioLegend	2
CD8	PerCP-Cy™5.5	RPA-T8	1:250	BioLegend	2
CD11b	Brilliant Violet 711™	ICRF44	1:50	BioLegend	1
CD14	FITC	MEM-18	1:10	ImmunoTools	1
CD16	APC-Cy™7	3G8	1:50	BioLegend	1
CD20	Alexa Fluor® 700	2H7	1:50	BioLegend	1
	Alexa Fluor® 488	2H7	1:2000	BioLegend	2
CD25 (IL-2RA)	Brilliant Violet 650™	BC96	1:250	BioLegend	2
CD45RA	Brilliant Violet 605™	HI100	1:300	BioLegend	2
CD45RO	Brilliant Violet 785™	UCHL1	1:100	BioLegend	2
CD56	PE-Cy™7	NCAM16.2	1:100	BD Biosciences	1
	Alexa Fluor® 488	NCAM1	1:50	BD Biosciences	2
CD123	Brilliant Violet 605™	9F5	1:1600	BD Biosciences	1
CD127	PE-Cy™7	A019D5	1:250	BioLegend	2
CD133	PE	W6B3C1	1:200	BD Biosciences	1
CD141	BB700	1A4	1:800	BD Biosciences	1
CD152 (CTLA4)	Brilliant Violet 421™	BNI3	1:50	BD Biosciences	2
CD161	PE	DX12	1:50	BD Biosciences	2
CD163	Brilliant Violet 650™	GHI/61	1:50	BD Biosciences	1
CD195 (CCR5)	Brilliant Violet 711™	3A9	1:50	BD Biosciences	2
CD197 (CCR7)	APC	G043H7	1:50	BioLegend	2
CD206	Brilliant Violet 421™	19.2	1:50	BD Biosciences	1
CD279 (PD-1)	Brilliant Violet 510™	EH12.2H7	1:100	BD Biosciences	2
HLA-DR	PE-CF594	G46-6	1:100	BD Biosciences	1
TCR Vα7.2	PE-Dazzle™594	3C10	1:50	BioLegend	2

Supplementary Figures

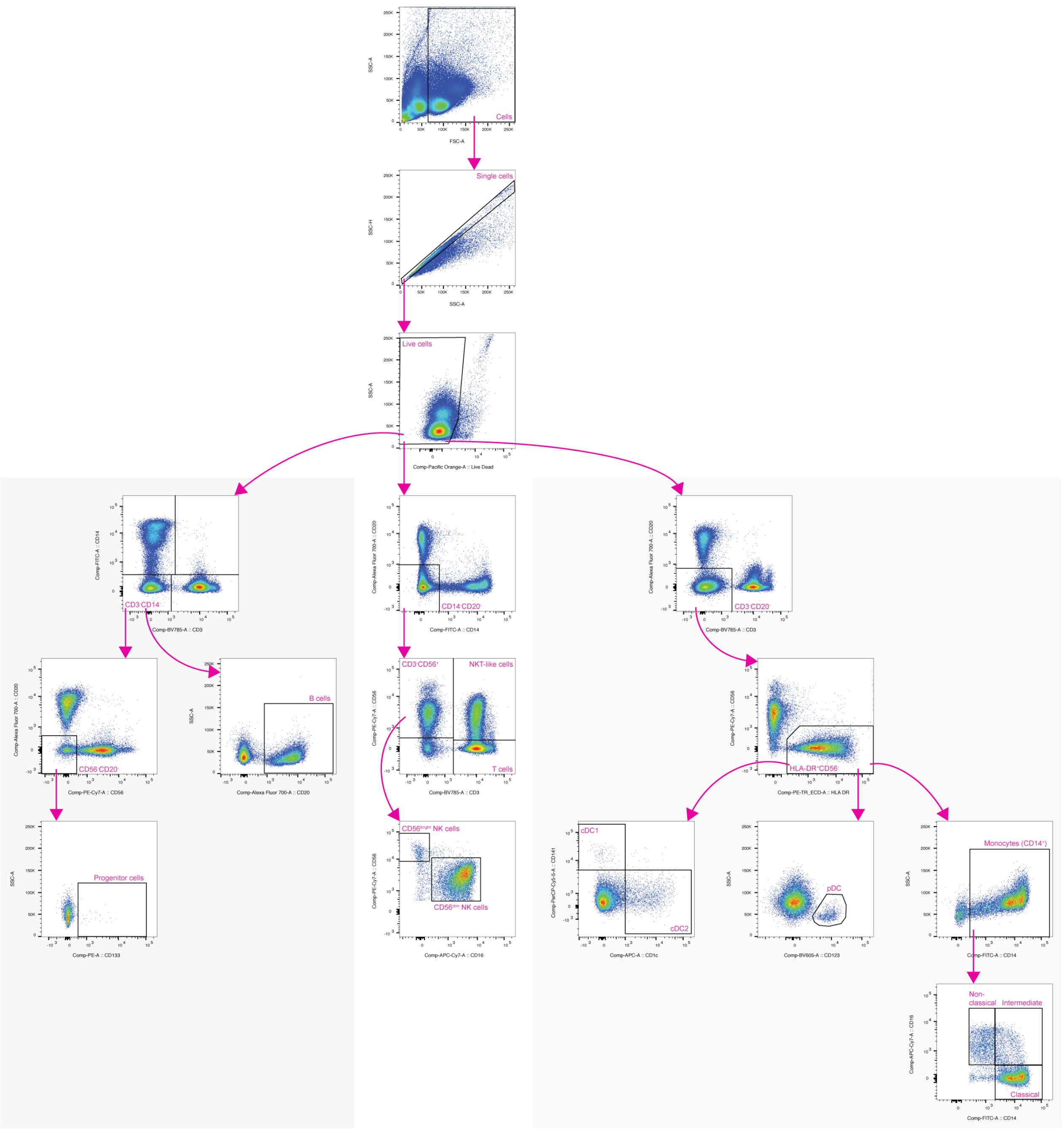

Supplementary Figure 1. Representative gating strategy used in the analysis of human PBMCs with flow cytometry panel 1. Intact cells were gated based on forward scatter area (FSC-A) and side scatter area (SSC-A). Single cells were identified based on their SSC-A and side scatter height (SSC-H) properties. Pacific orange was used to discriminate live from dead cells. The different cell types were then identified by using various combinations of cell surface markers tagged with fluorochromes.

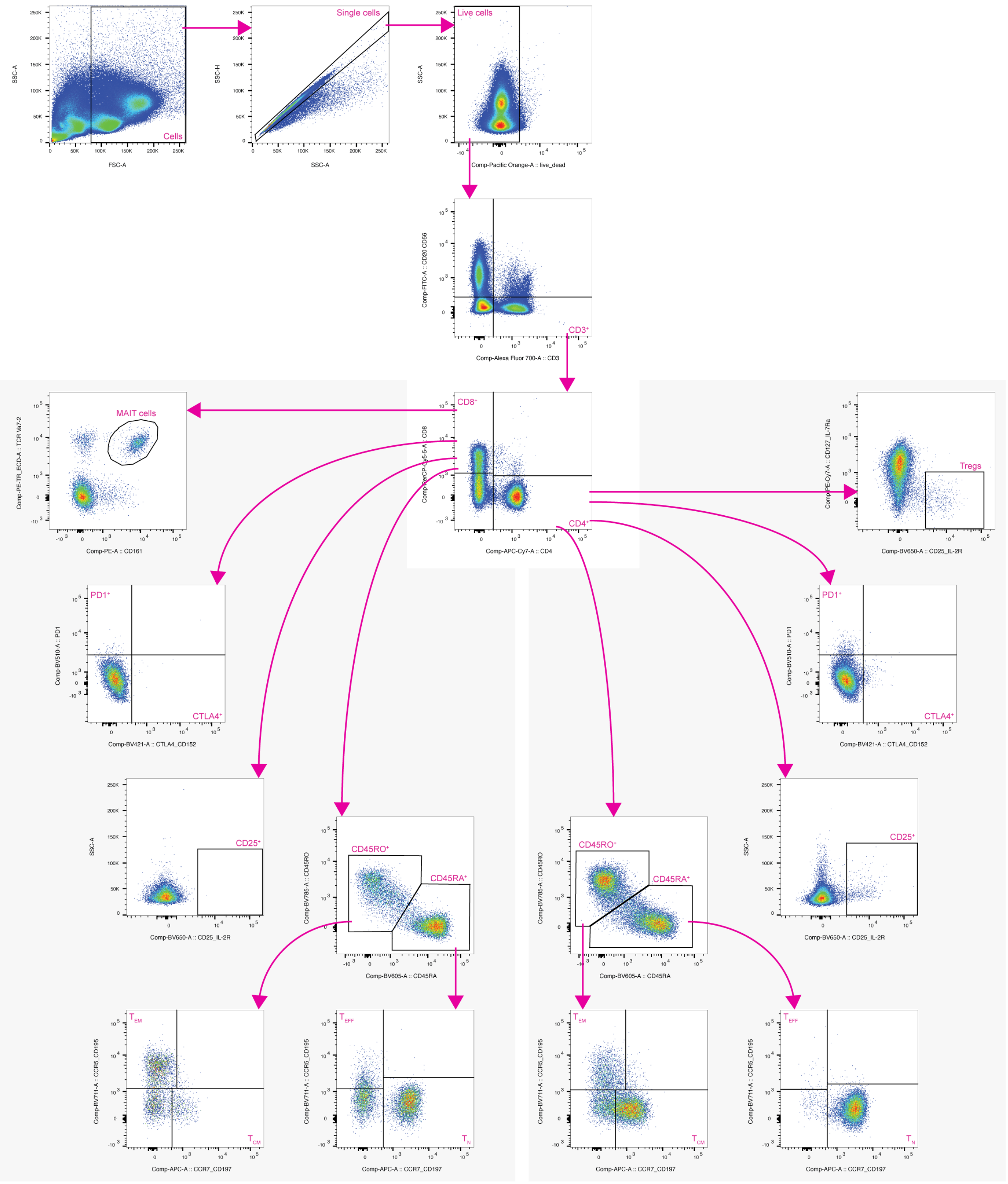

Supplementary Figure 2. Representative gating strategy used in the analysis of human PBMCs with flow cytometry panel 2. Intact cells were gated based on forward scatter area (FSC-A) and side scatter area (SSC-A). Single cells were identified based on their SSC-A and side scatter height (SSC-H) properties. Pacific orange was used to discriminate live from dead cells. T cells were defined as $CD3^{+}CD20^{-}CD56^{-}$. The different T cell types were then identified by using various combinations of cell surface markers tagged with fluorochromes. T_N...Naïve T cells; T_{EFF}...Effector T cells; T_M...Central memory T cells; T_{EM}...Effector memory T cells.

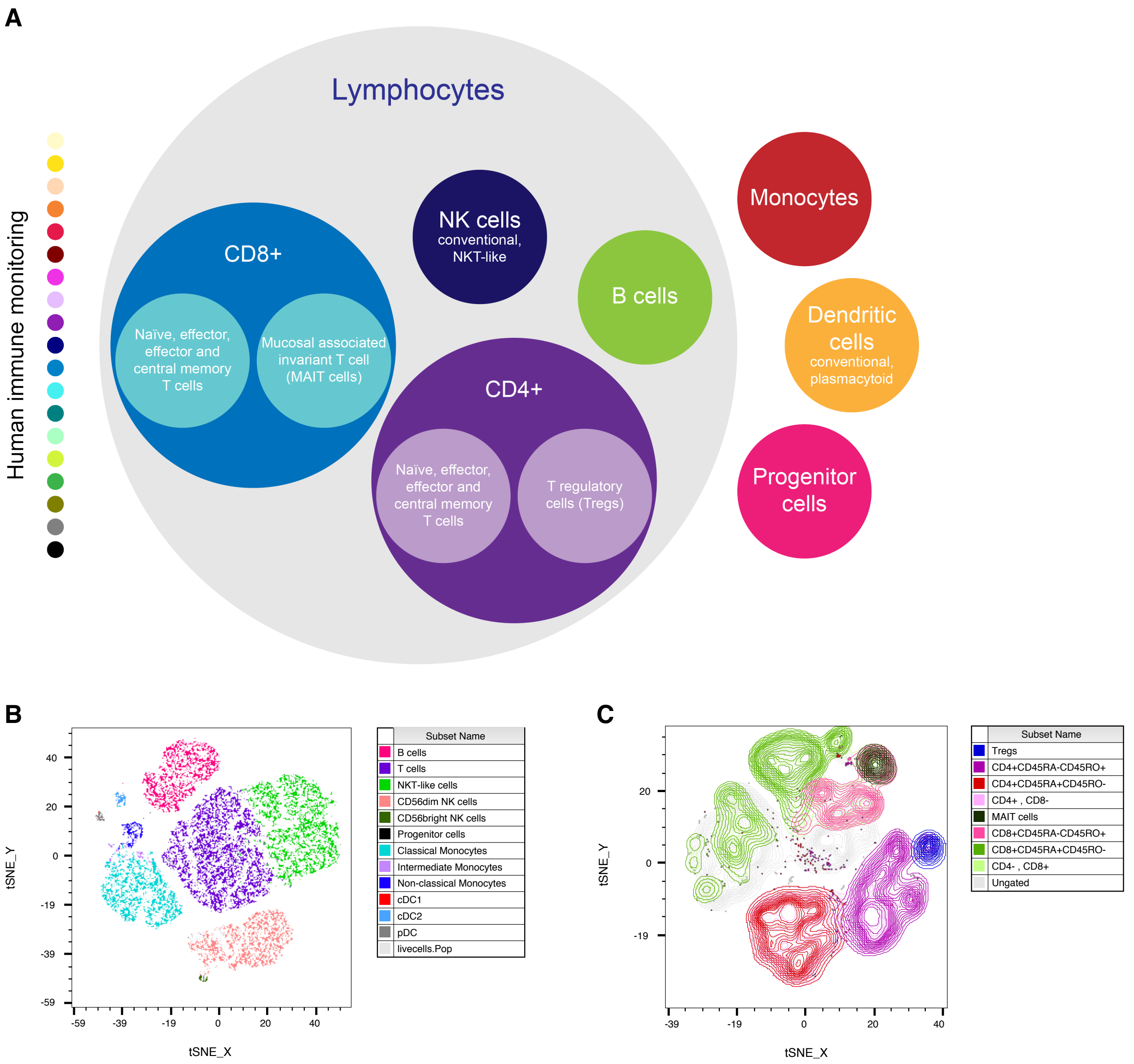

Supplementary Figure 3: Identified cell populations using two flow cytometry panels. (A) Summary of cell populations we are able to identify with our two carefully designed flow cytometry panels. Figure adapted and modified from Fluidigm. **(B)** Unsupervised gating using the tSNE function revealed the cell populations identified in single live cells using panel 1. One representative patient is shown (20000 cells). **(C)** tSNE plots showing the immune cell populations identified in single live T cells ($CD3^{+}CD20^{-}CD56^{-}$) applying panel 2. One representative patient is shown (20000 cells).

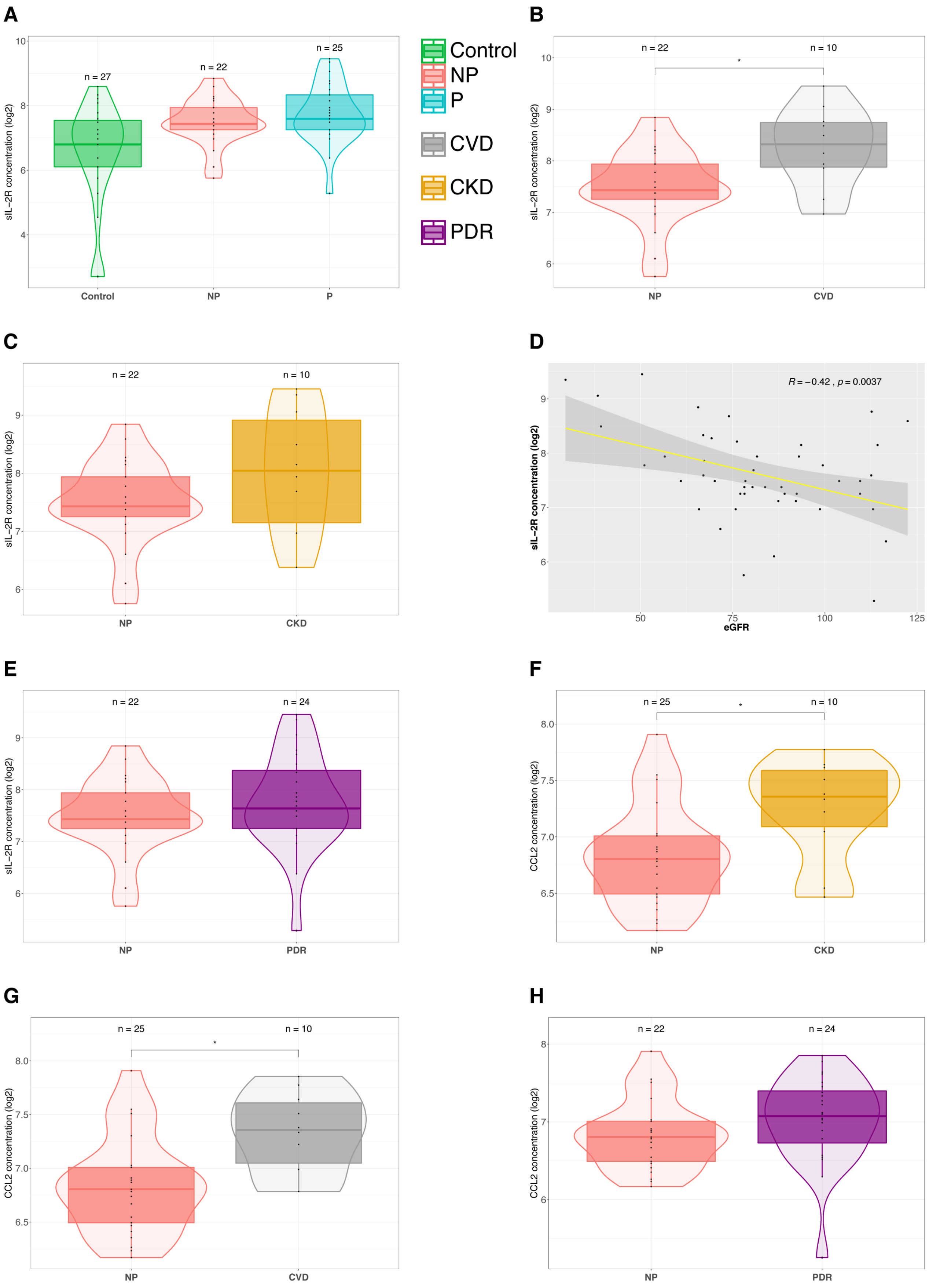
A
sIL−2R concentration (log2)
n = 27
n = 22
n = 25
Control
NP
P
Control
NP
P
CVD
CKD
PDR
B
sIL−2R concentration (log2)
n = 22
n = 10
NP
CVD
C
sIL−2R concentration (log2)
n = 22
n = 10
NP
CKD
D
R = −0.42 , p = 0.0037
sIL−2R concentration (log2)
eGFR
E
sIL−2R concentration (log2)
n = 22
n = 24
NP
PDR
F
CCL2 concentration (log2)
n = 25
n = 10
NP
CKD
G
CCL2 concentration (log2)
n = 25
n = 10
NP
CVD
H
CCL2 concentration (log2)
n = 22
n = 24
NP
PDR

Supplementary Figure 4. Plasma cytokine levels in DIALONG patients with type 1 diabetes (T1D) with different diabetes complications. (A) Patients with T1D from the DIALONG cohort had slightly elevated sIL-2R plasma levels in progressors (P) compared to non-progressors (NP). **(B)** Subgrouping of progressors by complication type showed significantly increased sIL-2R plasma levels in patients with T1D with cardiovascular disease (CVD) compared to NPs. **(C)** Plasma sIL-2R was slightly higher in patients with chronic kidney disease (CKD) compared to NPs. **(D)** Estimated glomerular filtration rate (eGFR) correlated negatively with sIL-2R in patients with T1D. **(E)** Patients with proliferative diabetic retinopathy (PDR) had slightly higher levels of sIL-2R compared to NPs. **(F)** CCL2 plasma levels in patients with T1D with CKD were significantly higher compared to NPs. **(G)** Progressors with CVD had significantly elevated CCL2 levels in comparison to NPs. **(H)** CCL2 was slightly higher in patients with PDR compared to NPs (The Mann-Whitney U test was used in the comparison between the different groups. Pearson correlation formula was used to investigate associations between variables. *$p < 0.05$, **$p < 0.01$, and ***$p < 0.001$).

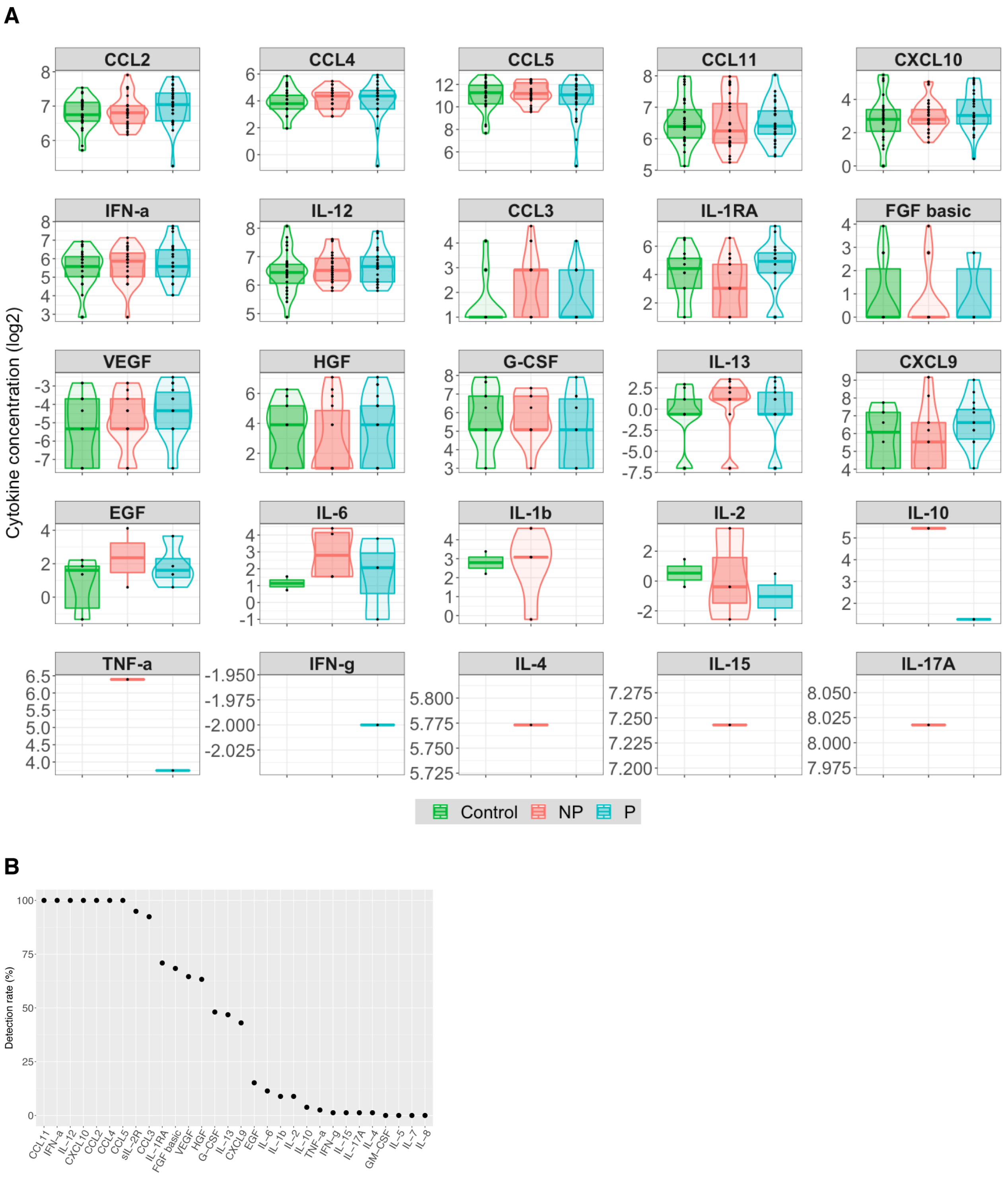

Supplementary Figure 5. Plasma cytokine levels in DIALONG healthy controls and patients with type 1 diabetes (T1D). (A) A 30-plex Luminex screening was performed in 79 DIALONG participants. All apart from sIL-2R and the undetected cytokines (GM-CSF, IL-5, IL-7, IL-8) are depicted in violin plots. No significant differences were observed between healthy controls, patients with T1D with complications (Progressors, P) and without complications (Non-progressors, NP) in these cytokines. **(B)** The detection rates from each investigated analyte are displayed. (The Mann-Whitney U test was used in the comparison between the different groups.).

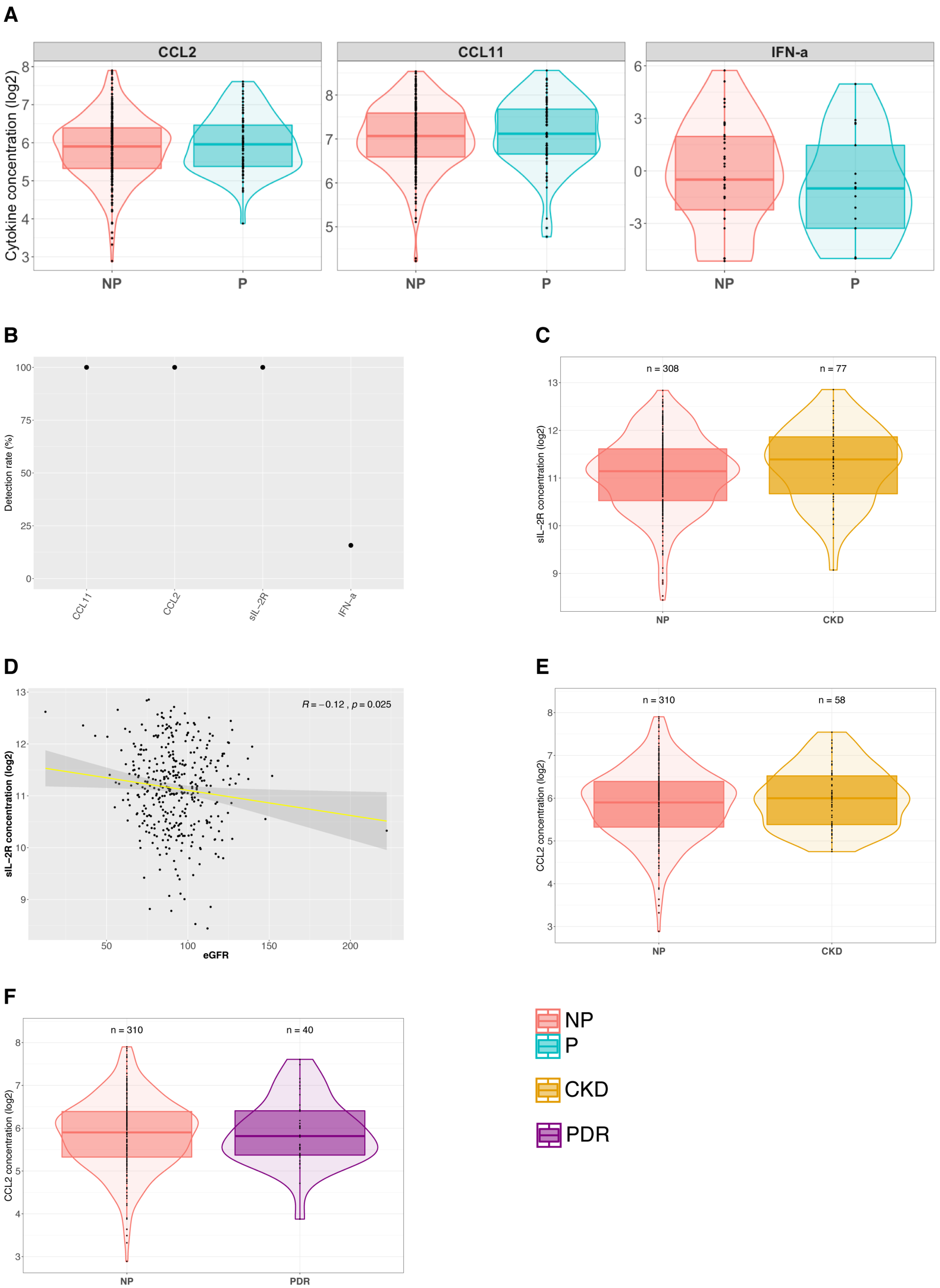

A
CCL2
CCL11
IFN-a
Cytokine concentration (log2)
NP
P
B
Detection rate (%)
CCL11
CCL2
sIL−2R
IFN−a
C
n = 308
n = 77
sIL−2R concentration (log2)
NP
CKD
D
R = −0.12 , p = 0.025
sIL−2R concentration (log2)
eGFR
E
n = 310
n = 58
CCL2 concentration (log2)
NP
CKD
F
n = 310
n = 40
CCL2 concentration (log2)
NP
PDR
NP
P
CKD
PDR

Supplementary Figure 6. Plasma cytokine levels in PROLONG patients with type 1 diabetes (T1D) with different diabetes complications. (A) A 4-plex Luminex screening was performed in 394 PROLONG participants. All apart from sIL-2R are depicted in violin plots. No significant differences were observed between PROLONG patients with T1D with complications (Progressors, P) and without complications (Non-progressors, NP) in these cytokines. **(B)** The detection rates from each investigated cytokine are displayed. **(C)** Subgrouping of progressors by complication type showed slightly increased sIL-2R plasma levels in patients with chronic kidney disease (CKD) compared to NPs. **(D)** Estimated glomerular filtration rate (eGFR) correlated negatively with sIL-2R. **(E)** There was no difference in CCL2 plasma levels between progressors with CKD and NPs. **(F)** Patients with proliferative diabetic retinopathy (PDR) and NPs showed similar levels of CCL2. (The Mann-Whitney U test was used in the comparison between the different groups. Pearson correlation formula was used to investigate associations between variables.).

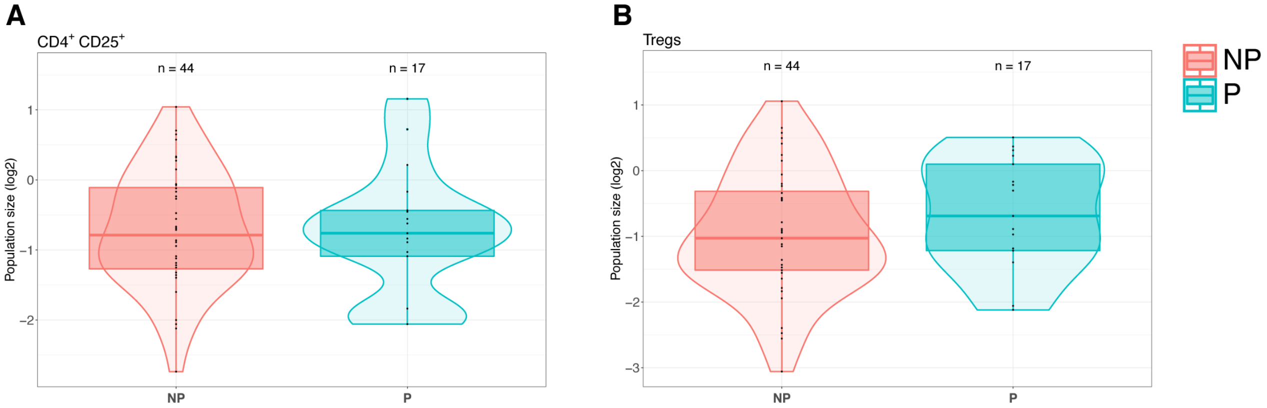

Supplementary Figure 7. Patients with type 1 diabetes (T1D) with complications do not display a difference in $CD25^{+}$ T cells and Tregs. (A) Patients with T1D with complications (Progressors, P) and without complications (Non-progressors, NP) showed similar levels of $CD3^{+}CD4^{+}CD25^{+}$ T cells. **(B)** We observed no differences in the Treg populations size between NPs and progressors. (For the comparison between the different groups, multiple linear regression was applied and adjusted for the age and sex covariates.).
